# Meta-analysis of incidence and risk of severe adverse events and fatal adverse events with crizotinib monotherapy in patients with *ALK*-positive NSCLC

**DOI:** 10.18632/oncotarget.18536

**Published:** 2017-06-17

**Authors:** Qian Zhu, Hao Hu, Feng Jiang, Chang Ying Guo, Xiong Wen Yang, Xi Liu, Yu Kang Kuang

**Affiliations:** ^1^ Department of Biotherapy, Sun Yat-sen University Cancer Center, Guangzhou 510060, Guangdong, China; ^2^ Department of Thoracic Surgery, Medical College of Nanchang University, Nanchang 330000, Jiangxi, China; ^3^ Department of Thoracic Surgery, Jiangxi Province Tumor Hospital, Nanchang 330006, Jiangxi, China; ^4^ Department of Lung Cancer Center, First People's Hospital Chenzhou, Chenzhou 423000, Hunan, China

**Keywords:** crizotinib, severe adverse effects, fatal adverse effects, non-small cell lung cancer, anaplastic lymphoma kinase

## Abstract

**Background:**

Numerous clinical trials show crizotinib has promising efficacy for anaplastic lymphoma kinase (*ALK*) positive non-small cell lung cancer (NSCLC) patients which trigger the substitution of traditional chemotherapy to be the current standard first-line treatment for these patients. Conversely, few reports systematically analyze toxicity of crizotinib. Hence, we performed a first meta-analysis to determine the risk of crizotinib-related severe adverse events (SAEs) and fatal adverse events (FAEs) in *ALK* positive NSCLC patients.

**Materials and Methods:**

A systematic literature search was conducted through December 2016 to identify clinical trials that reported crizotinib monotherapy in ALK-positive NSCLC patients. Data on crizotinib-related SAEs and FAEs were extracted from each study and pooled to determine the overall incidence and risk. Random-effects or fixed-effects models were conducted to calculate the summary incidence, relative risk (RR), and 95% CIs on basis of the heterogeneity of included studies.

**Results:**

1,924 patients from 11 clinical trials were included. The overall incidence of SAEs and FAEs with crizotinib was 19.9% (95% CI, 14.1% to 23.7%; *P* < 0.001) and 1.4% (95% CI, 0.9% to 2.1%; *P* < 0.001), respectively. Meanwhile, Asian patients have lower incidence of SAEs (11.5%, 95% CI: 7.9% to 16.5%). However, significant differences of SAEs (RR: 0.97, 95% CI, 0.79 to 1.18; *P* = 0.76) and FAEs (RR: 2.24, 95% CI, 0.49 to 10.30; *P* = 0.30) were not detected between crizotinib monotherapy and chemotherapy.

**Conclusions:**

Crizotinib may not increase the risk of SAEs and FAEs in patients with *ALK* positive NSCLC compared with chemotherapy.

## INTRODUCTION

Lung cancer is the leading cause of cancer morbidity and mortality in the world [1]. Approximately 85% of lung cancer cases are characterized as non-small cell lung cancer (NSCLC) cases [2] and anaplastic lymphoma kinase (*ALK*) positive is therein non-negligible, occurring in 2 to 7% of all NSCLC [3]. Crizotinib, a multiple small-molecule inhibitor of ALK, mesenchymal-epithelial transition (MET) and c-ros oncogene 1 (ROS1), was first approved in 2011 by the United States (US) Food and Drug Administration (FDA) for treatment of patients with local advanced or metastatic *ALK*-positive NSCLC based on the results of early phase clinical trials [4].

Now, crizotinib has become a recommended standard of care for patients with *ALK*-positive NSCLC according to the National Comprehensive Cancer Network guideline [5]. Meanwhile, it is usually reported that crizotinib is generally well tolerated in patients with *ALK*-positive NSCLC, with most treatment-related adverse events of a grade 2 or less, including gastrointestinal disturbances and visual events [6]. Other toxicities have been reported and mainly include peripheral edema, dizziness, fatigue and decreased appetite [7].

However, the incidence and risk of SAEs and FAEs are frequently overlooked during the treatment decision-making process, which have grave consequences to the patient, family and society [8]. Moreover, crizotinib-related SAEs and FAEs have been noted in clinical practice and clinical trials [9–12]. Unfortunately, few reports systematically analyze severe and fatal toxicity of crizotinib. Hence, we performed this meta-analysis to estimate the incidence and risk of SAEs and FAEs with crizotinib among *ALK*-positive NSCLC patients.

## MATERIALS AND METHODS

### Study strategy

In December 2016, we performed an electronic search of the Web of Science, EMBASE, PubMed, and Cochrane Library databases. Meanwhile, we searched abstracts presented at major meetings from the American Society of Clinical Oncology (ASCO), the European Society for Medical Oncology (ESMO) and the World Lung Cancer Conference (WCLC). An independent search of relevant reviews and meta-analyses associated with crizotinib was also done to ensure no studies were missed. The following key word was used: crizotinib. We limited language to English, but the publication years were not limited. Finally, reference lists of original papers and review papers were also scanned. We contacted the corresponding authors of some studies for further information if necessary. Our study was managed based on the Preferred Reporting Items for Systematic Reviews and Meta-analyses (PRISMA) guidelines [13].

### Study selection

The inclusion criteria were as follows: (a) crizotinib monotherapy in clinical trials; (b) treatment-related SAEs (grade 3/4) and FAEs (grade 5) were reported; (c) pathologic confirmation of *ALK*-positive NSCLC; and (d) publication language is English. If the articles were based on the same trial, the latest and the most complete data only were used for this analysis. Two investigators evaluated the articles for relevance independently.

### Exclusion criteria

Reviews, editorials, case reports were excluded. The aim of our study was to investigate incidence and risk of crizotinib monotherapy among adult patients with *ALK* positive NSCLC. We thus excluded studies involving pediatric patients, patients without *ALK* positive NSCLC, trials that combination with crizotinib and other therapy in the intervention and/or control cohorts.

### Study quality assessment

Two investigators (Q. Zhu and H. Hu) independently assessed risk of bias in randomized control trials (RCTs) using the Cochrane collaboration’s tool for assessing risk of bias [14]. Two investigators independently assessed each study under five main headings for risk of bias. Similarly, two investigators (F. Jiang and C Y. Guo) assessed the full texts of non-randomized clinical trials (NRCTs) using the 9-point Newcastle Ottawa scale (NOS) [15]. Each study was independently evaluated by two investigators based on eight items, categorized into three broad perspectives including selection, comparability and outcome for cohort studies or exposure for case-control studies. Studies with a score of 7 or greater were considered as high quality. Disagreements were resolved by discussion or through consultation with the senior reviewer.

### Data extraction

All articles were first catalogued (article title, author names and year of publication) before selection. The abstracts of the articles were evaluated independently by two investigators (X W. Yang and X. Liu). Outcomes were pooled for the occurrence of crizotinib-related SAEs and/or FAEs. Data were pooled and transferred into a standard electronic form. Discrepancies, when identified, were solved by discussion until a consensus was reached. Any final decision regarding the eligibility of a study and data were extracted by the principal investigator (Y K. Kuang).

### Clinical endpoints and statistical analysis

All statistical analysis was performed with Comprehensive Meta-analysis software, version 3 (Biostat, Englewood, USA). SAEs and FAEs, extracted from the safety profile in each trial with crizotinib monotherapy in *ALK*-positive NSCLC, were applied to clinical end points and noted according to version 3 or 4 of the Common Terminology Criteria for Adverse Events (CTCAE). Data on the number of patients with SAEs and FAEs, as well as the number of patients receiving crizotinib were extracted from the publications of the selected studies for the calculation of incidence and the proportion of patients with SAEs and FAEs and 95% confidence intervals (CIs) were deduced for each study. A classic half-integer continuity correction for the calculation of incidence and relative risk (RR) was used while zero events were reported in the crizotinib or control cohort. To compare adverse effect rates of crizotinib with control regimens, the RR was calculated. The pooled estimate for incidence and RRs were assessed with random-effects or fixed-effects model based on the heterogeneity of included studies. Assumption of homogeneity for the Cochrane Q statistic with values of *P* < 0.10 was considered to be invalid. Cochran’s Q test was used to assess between-study differences and the inconsistency was quantified with the I^2^ statistic. When the heterogeneity was in-existent, fixed-effects model was used to pool the summary incidence and RRs otherwise random effects model was used.

To explore the possible reasons for any observed heterogeneity, we performed the following pre-specified subgroup analyses and meta-regression: study design (prospective versus retrospective), ethnicity (Asian versus Multi-race) and percentage of Asian patients. We tried to limit the number of subgroup analyses with suggestion in the Cochrane Handbook and no post-hoc subgroup analyses were performed [16]. Also, subgroup analyses were not conducted while heterogeneity was nonexistent. Begg’s [17] and Egger’s tests [18] along with the funnel plots were assessed publication bias for both SAEs and FAEs. All tests were two-tailed and *P* < 0.05 was considered as statistical significance.

## RESULTS

### Trial flow and study characteristics

**Figure 1:**
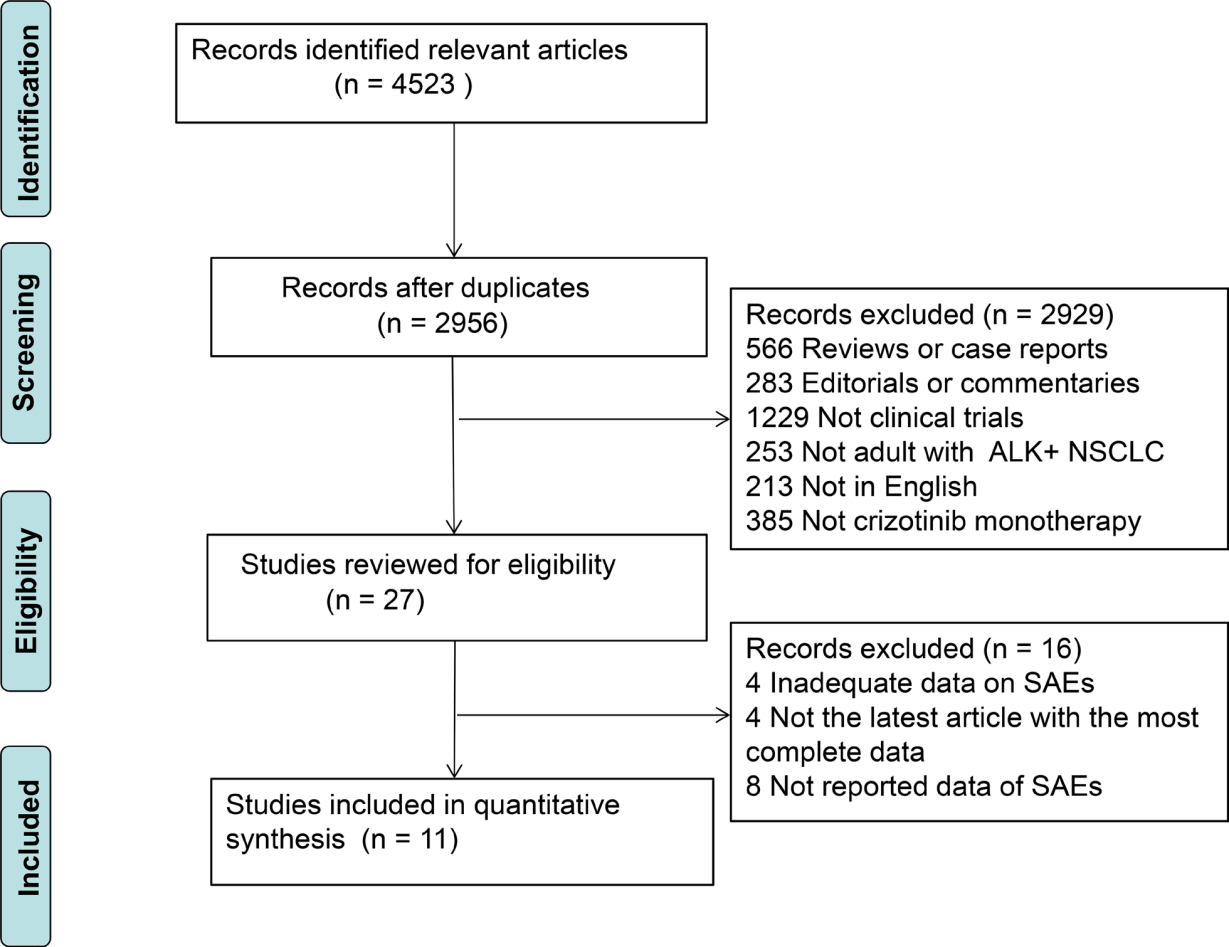
The flow diagram of meta-analysis *ALK*, anaplastic lymphoma kinase; NSCLC, non-small cell lung cancer; SAEs, severe adverse events.

Our literature search yielded a total of 4523 articles on crizotinib. After evaluating each publication, we identified 27 studies for eligibility. After further evaluation, sixteen studies were excluded (four studies: inadequate data on severe AEs, four studies: not the latest article with the most complete data, eight studies: not reported data of SAEs and FAEs). The rest eleven studies met our inclusion criteria [6, 12, 19–27] (Figure 1). Five studies [6, 12, 20–22] were prospective and six studies [19, 23–27] were retrospective. A total of 1,924 patients were available for the meta-analysis. All the patients tend to be younger (median age: 42–57), adenocarcinoma (94%–100%) and advanced stage (stage III and/or IV). All the initial crizotinib dose and schedule of dosage was based on the US FDA guidelines (250 mg, orally, twice a day) and at least one dose of the treatment was utilized. The baseline characteristics of all studies were listed in Table 1.

**Table 1:** Primary characteristics of the selected studies

Study (Reference)	Year	Numbers (Safety)	Age (Median)	Ethnicity	A (%)	SAEs	FAEs	Line of therapy	Study design	PS ≥2 (%)	Clinical stage
D Ross Camidge [6]	2012	149	52 Y	Multi-race	97%	36	0	Mixed-line	prospective	12%	Stage III /IV
Alice T. Shaw [12]	2013	172	51 Y	Multi-race	95%	57	3	Second-line	prospective	9%	Stage III /IV
Yabing, Cao [19]	2014	40	42 Y	Asian	100%	6	0	Mixed-line	retrospective	NA	Stage III /IV
Benjamin J. Solomon [20]	2014	171	52 Y	Multi-race	94%	60	0	First-line	prospective	6%	Stage III /IV
Shaohua,Cui [21]	2015	72	55 Y	Asian	94.4%	10	1	Mixed-line	prospective	2.8%	Stage III /IV
PROFILE1005 [22]	2015	1066	52.2 Y	Multi-race	95%	425	15	Mixed-line	prospective	NA	Stage III /IV
Yan, Wang [23]	2015	53	50 Y	Asian	98%	3	0	Mixed-line	retrospective	21%	Stage III /IV
Shaohua, Cui [24]	2016	56	55 Y	Asian	100%	8	1	Mixed-line	retrospective	2%	Stage III /IV
Puyuan Xing [25]	2016	34	51.5 Y	Asian	NA	2	0	Mixed-line	retrospective	2.9%	Stage IV
Tatsuya Yoshida [26]	2016	35	57 Y	Asian	100%	3	0	Mixed-line	retrospective	14%	Stage III /IV
Quan, Zhang [27]	2016	7	53 Y	Asian	100%	1	0	First-line	retrospective	0%	Stage IV

Y: years; A: Adenocarcinoma; SAEs: Severe adverse events. FAEs: Fatal adverse events. NA: Not available. PS: Performance Status.

### Study quality assessment and risk of bias

No major flaw was observed in assessment of risk of bias in RCTs. However, a common flaw was the absence of expected blinded intervention (more details were summarized in the Supplementary Table 1). We summarized the methodological quality of all the NRCTs (excluding PROFILE 1005 trial) in the Supplementary Table 2. The NOS results showed that the average overall score was 6.75 (range 6–9).

### Incidence of SAEs

**Figure 2:**
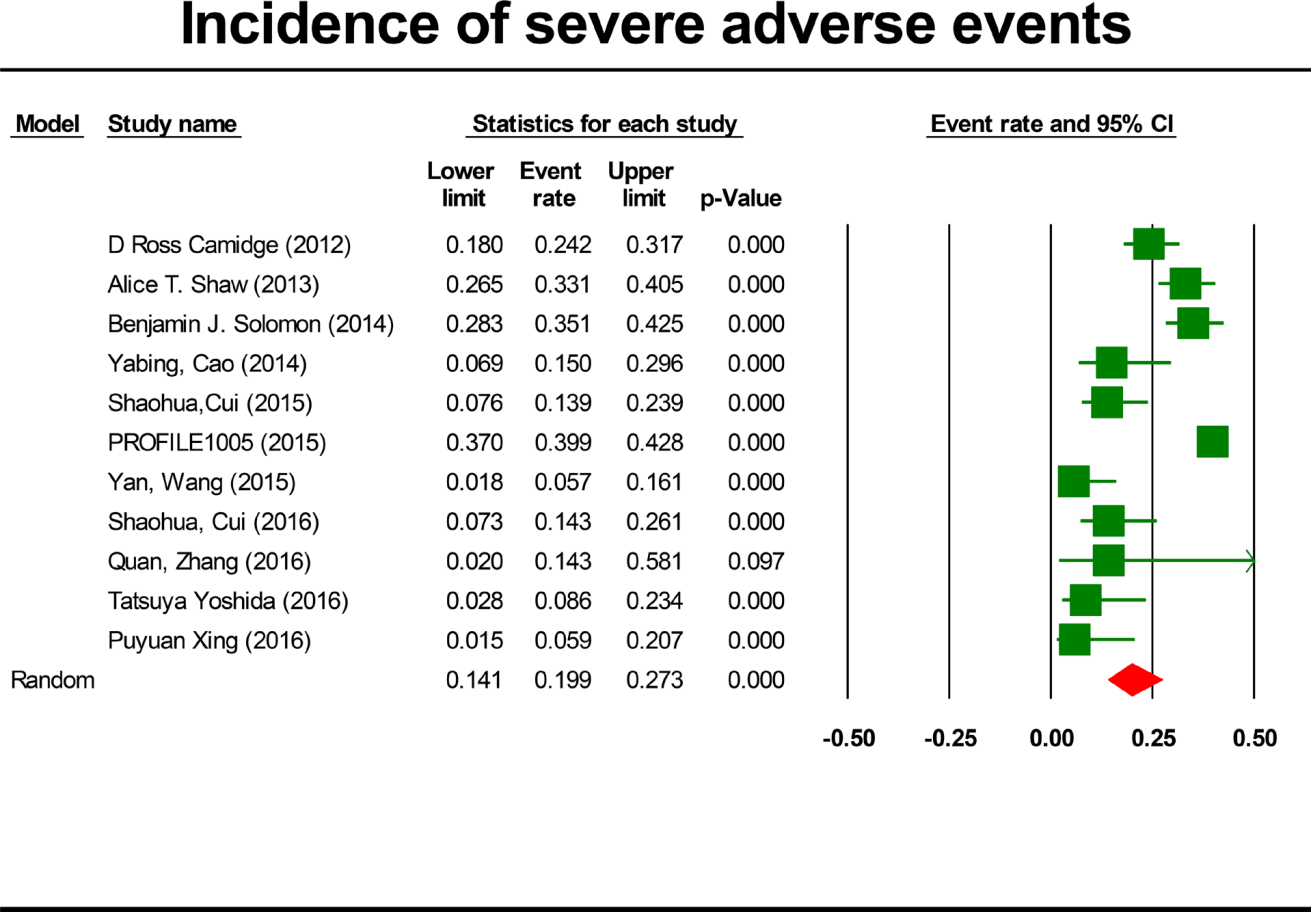
Forest-plot of the overall incidence of crizotinib-related severe adverse events

For the incidence of SAEs, all studies were included, representing a total of 1,924 patients. By using a random effects model (heterogeneity test: Q = 79.01; I^2^ = 87.34%; *P* < 0.001), the incidence of crizotinib-related SAEs was determined to be 19.9% (95% CI, 14.1% to 23.7%, *P* < 0.001; Figure 2). The incidence of SAEs was highest in the PROFILE 1005 trial [22] (39.9%; 95% CI, 37.0% to 42.8%) and lowest in the Wang study [23] (5.7%; 95% CI, 1.8% to 16.1%). The incidence of SAEs varied significantly with study design (*P* < 0.001) and ethnicity (*P* < 0.001). The summary incidence rates were 30% (95% CI, 23.2% to 37.8%) for prospective study and 10.5% (95% CI, 6.4% to 16.8%) for retrospective study, respectively. Notably, the summary incidence rate was lower in Asian patients (11.5%, 95% CI: 7.9% to 16.5%) (Table 2). To further analyze the causes of heterogeneity, we performed meta-regression. There was significant correlation between the proportion of Asian patients and the incidence of SAEs (*P* < 0.001). Hence, the proportion of Asian patients can partly explain the heterogeneity between the trials.

**Table 2:** Heterogeneity in the incidence of crizotinib-related severe adverse events

		Incidence	95% CI	*P*-value for heterogeneity test
Study design	prospective study	30%	23.2%–37.8%	*P* < 0.001
	retrospective study	10.50%	6.4%–16.8%	
Ethnicity	Asian	11.50%	7.95–16.5%	*P* < 0.001
	Multi-race	33.60%	27.9%–39.9%	

No evidence of publication bias was detected for incidence of SAEs by the Begg’s (*P* = 0.12) but the Egger’s test (*P* < 0.001).

### Incidence of FAEs

**Figure 3:**
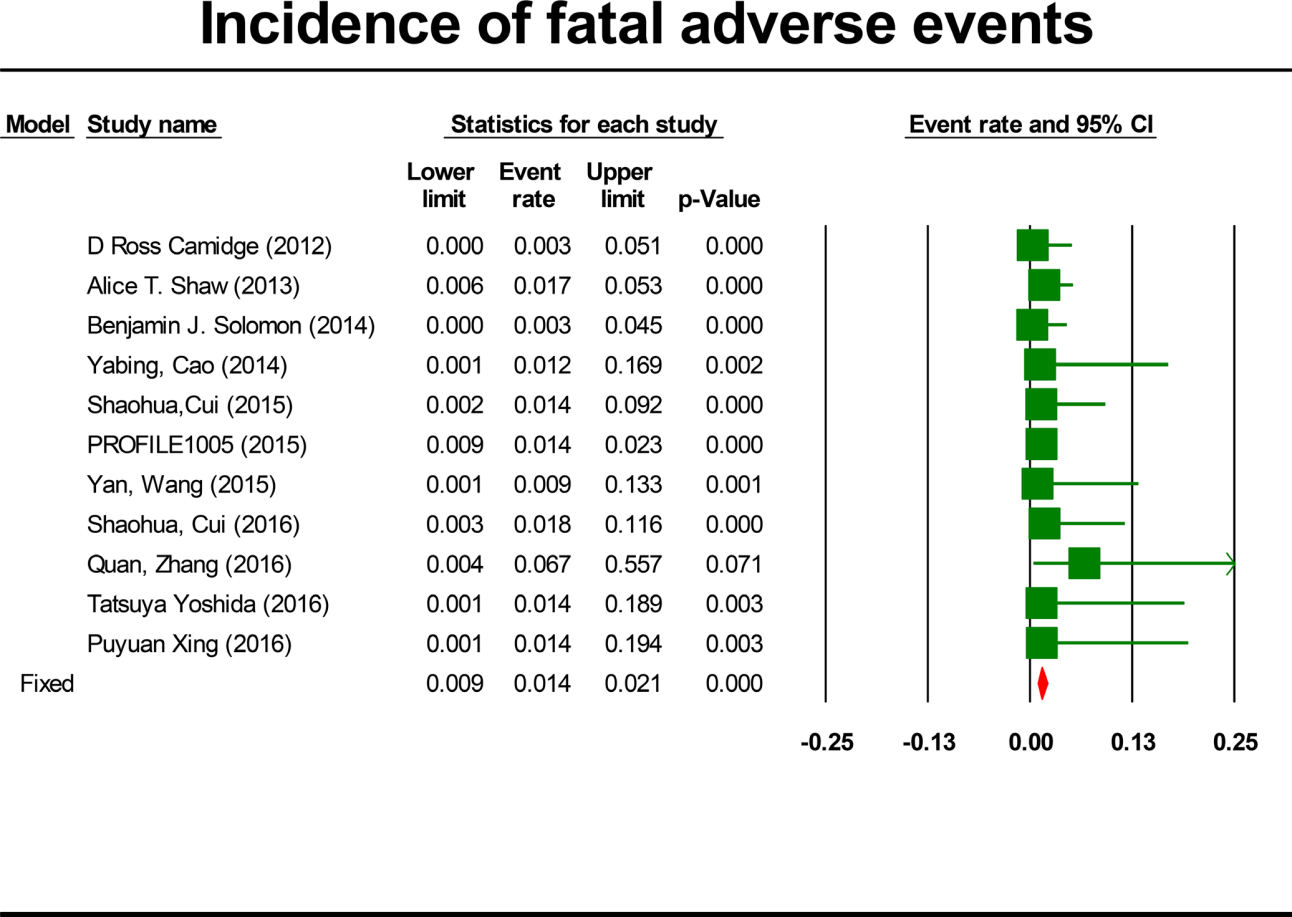
Forest-plot of the overall incidence of crizotinib-related fatal adverse events

For the incidence of FAEs, all these studies were included, representing a total of 1,924 patients. By using a fixed effects model (heterogeneity test: Q = 3.79; I^2^ = 0%; *P* = 0.956), the incidence of FAEs due to crizotinib was determined to be 1.4% (95% CI, 0.9% to 2.1%, *P* < 0.001; Figure 3). The incidence of FAEs was highest in the Zhang study [27] (6.7%) and lowest in the Camidge study [6] and Solomon study [20] (0.3%). No FAEs were observed in seven trials [6, 19, 20, 23, 25–27]. Because heterogeneity was not observed, subgroup analyses were not done for FAEs. To account for any possible clinical heterogeneity not detected by statistical tests, random-effects model was also used to pool the data: the incidence and 95% CI remained unchanged.

No evidence of publication bias was detected for incidence of SAEs by either the Begg’s (*P* = 0.75) or the Egger’s test (*P* = 0.60).

### RR of SAEs and FAEs

Four studies comparing crizotinib with chemotherapy were included to provide the RR of toxicity profile results. In the crizotinib group, all the initial crizotinib dose and schedule of dosage was 250 mg, orally, twice a day. In the chemotherapy group, platinum-based double-agent chemotherapy was used in all included studies except one study [12] with single-agent chemotherapy. The overall incidence of chemotherapy-related SAEs was determined to be 26.5% (95% CI, 16.6% to 39.4%, *P* = 0.001; Supplementary Figure 1).

**Figure 4:**
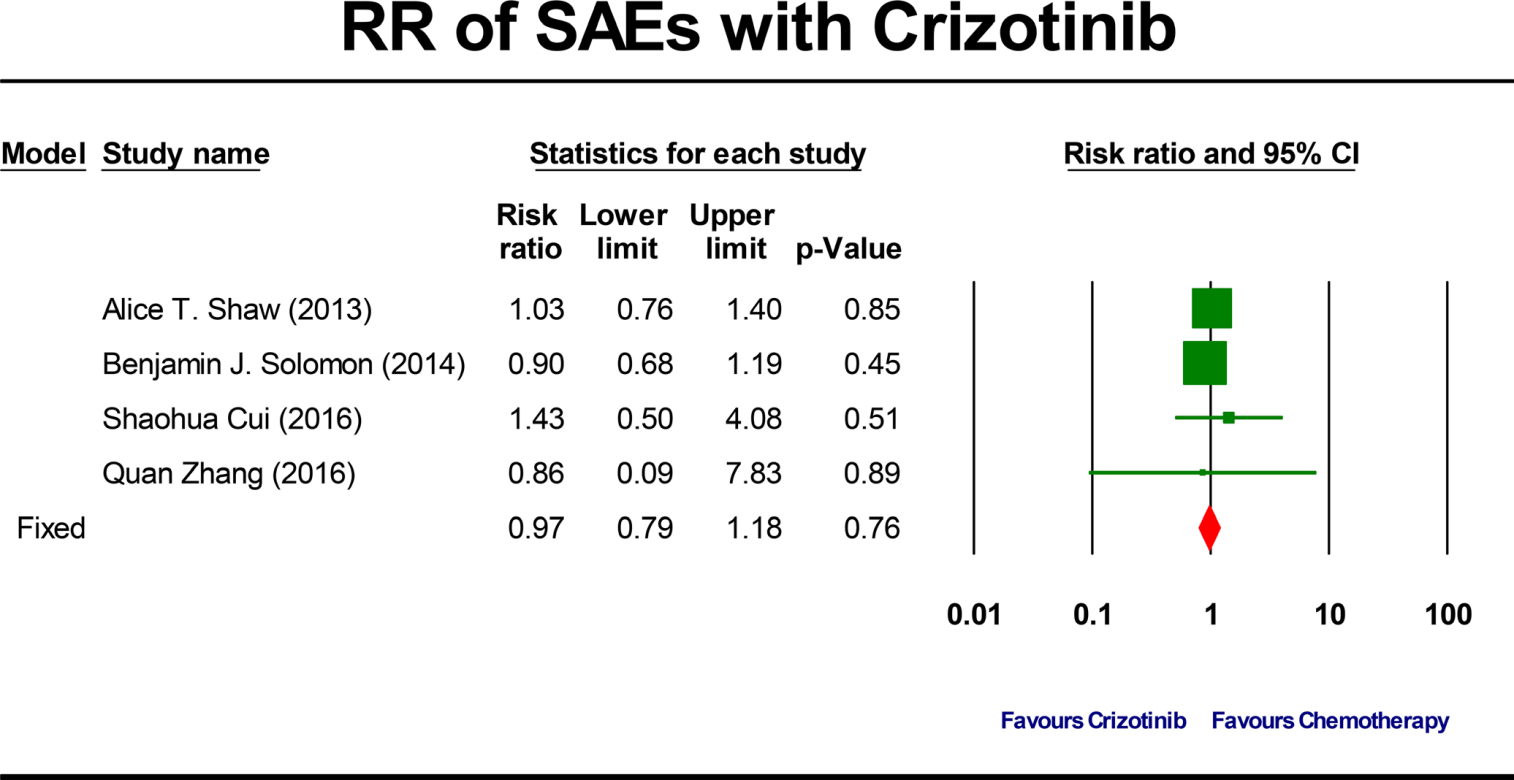
Forest-plot of the relative risk of severe adverse events (SAEs) associated with crizotinib versus chemotherapy

The summary RR of developing a crizotinib-related SAEs was 0.97 ( 95% CI, 0.79 to 1.18, *P* = 0.76; Figure 4). This estimate was acquired by using fixed-effects model because no significant heterogeneity was detected (Q = 0.98, *P* = 0.81, I^2^ = 0.0%) and therefore subgroup analyses were not done. To account for any possible clinical heterogeneity not detected by statistical tests, we also pooled the data using random-effects model: the RR and 95% CI remained unchanged.

**Figure 5:**
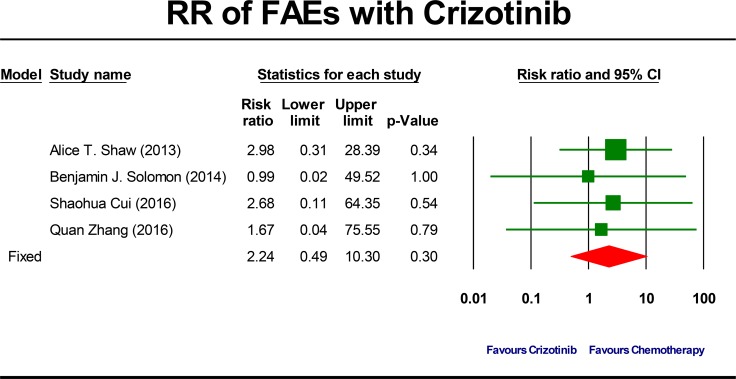
Forest-plot of the relative risk of fatal adverse events (FAEs) associated with crizotinib versus chemotherapy

The summary RR of developing a crizotinib-related FAEs was 2.24 ( 95% CI, 0.49 to 10.30, *P* = 0.30; Figure 5). This estimate was also acquired by using fixed-effects model because no significant heterogeneity was detected (Q = 0.27, *P* = 0.97, I^2^ = 0.0%) and therefore subgroup analyses were also not done. To account for any possible clinical heterogeneity not detected by statistical tests, we also pooled the data using random-effects model: the RR and 95% CI remained unchanged.

## DISCUSSION

Crizotinib has been the current standard first-line treatment for *ALK* positive NSCLC patients based on the result of the prospectively randomized PROFILE 1014 trial [20]. Although efficacy was quite important in clinical practice and clinical trials, safety profile was also notable. Up to now, this is the first and largest study to determine incidence and risk of crizotinib-related SAEs and FAEs in *ALK* positive NSCLC patients. The crude overall incidence of crizotinib-related SAEs and FAEs was 19.9% and 1.4%, respectively. Meantime, crizotinib use may not increase the risk of both SAEs (RR: 0.97, *P* = 0.76) and FAEs (RR: 2.24, *P* = 0.30) compared to chemotherapy. Of note, the risk of developing an FAE was more than two-fold higher in patients treated with crizotinib compared with patients treated in chemotherapy arms. However, no statistically significant difference was detected and the difference between two groups was probably affected by the fact that the duration of treatment in the crizotinib group was longer than those in the chemotherapy group and that more patients in the crizotinib group continued treatment beyond progression [12, 28]. Moreover, the occasional wide variation in the confidence interval of the risk was observed and may be ascribed to the limited small number of trials. Hence, the current findings did not produce adequate power to detect potentially relevant differences in FAEs between the two strategies.

It is noteworthy that severe adverse events (grade 3 or 4) defined are different with the definitions of serious adverse events, although the abbreviation was the same as “SAEs”. A serious adverse event was defined as follows: death; life-threatening; hospitalization or prolongation of existing hospitalization; results in persistent or significant disability/incapacity and so on [20]. Nonetheless, according to CTCAE, version 4.0, grade 3 and 4 were defined as “severe or medically significant but not immediately life-threatening; hospitalization or prolongation of hospitalization; disabling; limiting activities of daily living” and “life-threatening consequences”, respectively. Thus, it is different between severe adverse events and serious adverse events. For instance, the incidence of treatment-related serious adverse events in the PROFILE 1014 trial was 10.5% [29]. However, crizotinib-related SAEs in the PROFILE 1014 trial were mostly elevated aminotransferases (14%) and neutropenia (11%) [20]. These toxicities might be managed with dose interruptions or dose reductions and without life-threatening conditions or hospitalization [20].

Although the actual incidence of FAEs was relatively low at 1.4%, SAEs developed in as many as almost one-fifth of all patients receiving crizotinib. Treating cancer with modern targeted therapies is a double edged sword. Patients frequently incline to overestimate the benefit of treatment and underestimate the harms [30]. Therefore, the discussion of the adverse effects (especially for SAEs) between clinicians and patients prior to treatment was necessary in routine clinical practice. Without understanding of these adverse events properly, patients and clinicians will unable to proper judge the risk-benefit balance [8]. Hence, it is worth to notice these toxicity profiles in routine clinical practice and clinical trials.

It is remarkable that the incidence of crizotinib-related SAEs in Asian patients was lower (11.5%). It is consistent with the previous report, which estimated the differences in crizotinib pharmacokinetics between Asian and non-Asian patients, that Asian patients have lower incidence of SAEs [31]. However, it should be pointed out that all studies with only Asian patients included in our meta-analysis were retrospective. Moreover, as shown in Table 2, significant differences of incidence of SAEs were observed between prospective and retrospective studies in the exploratory subgroup analysis. Meanwhile, as shown in Supplementary Table 3, there were some differences in the safety assessment between prospective and retrospective studies. What is more, it was not reported that how an adverse event was assessed and what factors (physical examintation, documentation of adverse events or laboratory test) were included in most retrospective studies. Hence, bias and confounding in retrospective studies (for instance, lack of management of toxicity, selection bias) may bias the finding. Even so, random-effects model was used in the analysis of incidence of SAEs.

The ALK expresses in many tissues including eye, olfactory nerve, skins, tissue surrounding the esophagus, stomach and midgut [32]. Therefore, on-target anti-ALK effects in normal tissue play an important role in inducing side-effects during the treatment [6]. However, crizotinib is also a MET and ROS1 inhibitor. Thus, whether anti-MET effects, anti-ROS1 effects or other specific effects could also be contributing to these adverse events is still unclear.

Interestingly, with an increasing number of patients being treated with crizotinib, new and more adverse effects of crizotinib are coming to light such as hormonal and electrolyte abnormalities, grade IV hypersensitivity rashes [33]. Notably, crizotinib is a multiple small-molecule inhibitor of ALK, MET and ROS1. Liu et al. [34] suggested that the peripheral edema was more common in c-MET inhibition compared to crizotinib. As what mentioned before, this adverse event was observed significantly less in a Phase I study of ceritinib, as well as alectinib, which more specifically inhibits ALK without inhibition of c-MET [33]. Hence, it seems reasonable to hypothesize that more specific inhibitors, focused solely on the ALK tyrosine kinase, bring patients less toxicity with higher quality of life.

There have several potential limitations to this analysis. One obvious limitation is the tag of these adverse events as treatment-related or disease-related by the study investigator could lead to bias since SAEs and FAEs were not the primary endpoint of any of the trials included in this meta-analysis. Further, other limitations were that the unavailability of details of SAEs data from Noronha study [35] as well as the detection of publication bias in the meta-analysis of SAEs.

## CONCLUSIONS

Crizotinib may not increase the risk of SAEs and FAEs in *ALK* positive NSCLC patients compared with chemotherapy. Although generally tolerated, appropriate care should be provided in order to minimize its toxicity and the dose needs to be adjusted in case of necessary.

## SUPPLEMENTARY MATERIALS FIGURE AND TABLES


