# Supplementary material for: Meta-analysis of incidence and risk of severe adverse events and fatal adverse events with crizotinib monotherapy in patients with ALK-positive NSCLC
Source: Oncotarget. 2017 Jun 17;8(43):75372–80. doi: 10.18632/oncotarget.18536 (PMC5650427; doi:10.18632/oncotarget.18536)
Supplement: Supplementary file 1 [file oncotarget-08-75372-s001.pdf]

Meta-analysis of incidence and risk of severe adverse events and fatal adverse events with crizotinib monotherapy in patients with *ALK*-positive NSCLC

Supplementary Materials

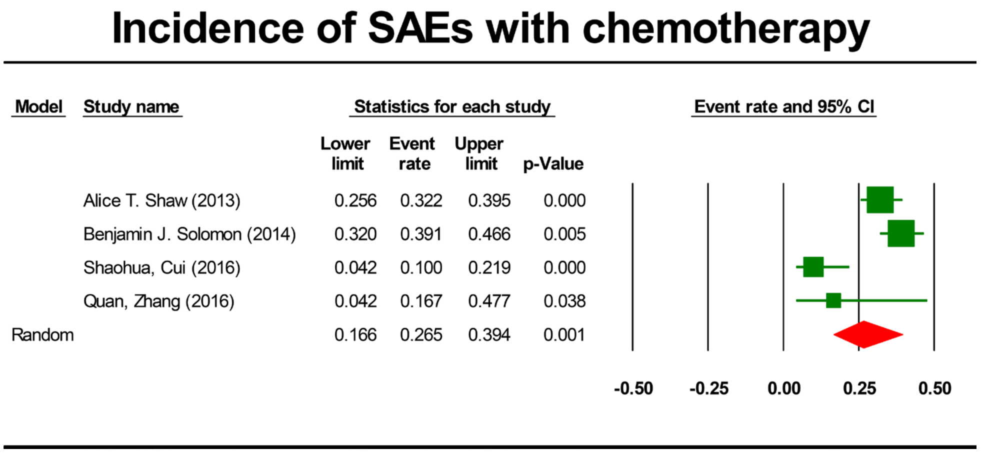

Supplementary Figure 1: Forest-plot of the overall incidence of chemotherapy-related severe adverse events.

Supplementary Table 1: Risk of bias in randomized controlled trials

Study	Selection bias	Performance bias	Detection bias	Attrition bias	Reporting bias	Other bias
Shaw, Alice T.[12]	Unclear	High	High	Low	Low	Low
Benjamin J. Solomon [20]	Unclear	High	High	Low	Low	Low

Supplementary Table 2: Nine-point newcastle ottawa scale scores for non-randomized clinical trials

Study (Reference)	Selection (Maximum of four stars)				Comparability (Maximum of two stars)	Outcome (Maximum of three stars)		
	Representativeness of the exposed cohort	Selection of the non exposed cohort	Ascertainment of exposure	Demonstration that outcome of interest was not present at start of study	Comparability of cohorts on the basis of the design or analysis	Assessment of outcome	Was follow-up long enough for outcome to occur	Adequacy of follow-up of cohorts
Camidge D [6]	*		*	*		*	*	*
Yabing, Cao [19]	*		*	*		*	*	*
Shaohua,Cui [21]	*		*	*		*	*	*
Yan, Wang [23]	*		*	*		*	*	*
Shaohua,Cui [24]	*	*	*	*	**	*	*	*
Puyuan Xing [25]	*		*	*		*	*	*
TatsuyaYoshida [26]	*		*	*		*	*	*
Quan, Zhang [27]	*	*	*	*	**	*	*	*

A study can be awarded a maximum number of four stars for the selection domain, maximum of two stars for the comparability domain and maximum of three stars for the outcome domain.

Supplementary Table 3: Details of safety assessment in the included studies

Study (Reference)	Study design	Safety assessment	Inclusion factors	Grade criterion (CTCAE)
D Ross Camidge [6]	prospective	at least every 2 weeks for the first 8 weeks of treatment and at least every 4 weeks thereafter until cycle 10, when visits every 8 weeks were permissible	physical examination, documentation of adverse events, and laboratory tests	version 3.0
Alice T. Shaw [12]	prospective	from the time the patient provided written informed consent until at least 28 days after the last dose of study drug	physical examination, documentation of adverse events , laboratory studies	version 4.0
Yabing, Cao [19]	retrospective {, #19573}	at least one treatment cycle	physical examination, vital signs, and laboratory studies	version 3.0
Benjamin J. Solomon [20]	prospective	from the first dose of study medication until 28 days after the last dose of study medication	physical examination, documentation of adverse events, laboratory studies	version 4.0
Shaohua,Cui [21]	prospective	at least twice in each treatment cycle	documentation of adverse events, ECG finding, and laboratory studies	version 3.0
PROFILE1005 [22]	prospective	NA	NA	version 4.0
Yan, Wang [23]	retrospective	NA	NA	version 4.0
Shaohua, Cui [24]	retrospective	at least twice per treatment cycle	documentation of adverse events, ECG finding, and laboratory studies	version 3.0
Puyuan Xing [25]	retrospective	NA	NA	version 3.0
Tatsuya Yoshida [26]	retrospective	NA	NA	version 4.0
Quan, Zhang [27]	retrospective	NA	NA	version 4.0

CTCAE: Common Terminology Criteria for Adverse Events. NA: Not available.
